# Uncovering the role of a positive selection site of wax ester synthase/diacylglycerol acyltransferase in two closely related *Stipa* species in wax ester synthesis under drought stress

**DOI:** 10.1093/jxb/eraa194

**Published:** 2020-04-20

**Authors:** Yunqiang Yang, Zhili Zhou, Yan Li, Yanqiu Lv, Danni Yang, Shihai Yang, Jianshuang Wu, Xiong Li, Zhijia Gu, Xudong Sun, Yongping Yang

**Affiliations:** 1 CAS Key Laboratory for Plant Diversity and Biogeography of East Asia, Kunming Institute of Botany, Chinese Academy of Science, Kunming, China; 2 Plant Germplasm and Genomics Center, Kunming Institute of Botany, Chinese Academy of Sciences, Kunming, China; 3 Institute of Tibetan Plateau Research at Kunming, Kunming Institute of Botany, Chinese Academy of Sciences, Kunming, China; 4 Functional Biodiversity, Dahlem Center of Plant Sciences, Free University of Berlin, Berlin, Germany; 5 University of Chinese Academy of Sciences, Beijing, China; 6 College of Life Sciences, Changchun Normal University, Changchun, China; 7 The James Hutton Institute, UK

**Keywords:** Drought, positive selection site, *Stipa* species, Tibetan Plateau, transcriptome, wax ester synthesis

## Abstract

Natural selection drives local adaptations of species to biotic or abiotic environmental stresses. As a result, adaptive phenotypic divergence can evolve among related species living in different habitats. However, the genetic foundation of this divergence process remains largely unknown. Two closely related alpine grass species, *Stipa capillacea* and *Stipa purpurea*, are distributed in different rainfall regions of northern Tibet. Here, we analyzed the drought tolerance of these two closely related *Stipa* species, and found that *S. purpurea* was more resistance to drought stress than *S. capillacea.* To further understand the genetic diversity behind their adaptation to drought environments, a comprehensive gene repertoire was generated using PacBio isoform and Illumina RNA sequencing technologies. Bioinformatics analyses revealed that differential transcripts were mainly enriched in the wax synthetic pathway, and a threonine residue at position 239 of WSD1 was identified as having undergone positive selection in *S. purpurea*. Using heterologous expression in the *Saccharomyces cerevisiae* mutant H1246, site-directed mutagenesis studies demonstrated that a positive selection site results in changes to the wax esters profile. This difference may play an important role in *S. purpurea* in response to drought conditions, indicating that *S. purpurea* has evolved specific strategies involving its wax biosynthetic pathway as part of its long-term adaptation to the Qinghai–Tibet Plateau.

## Teaser

The positive selection site of the wax ester synthase/diacylglycerol acyltransferase gene changed the wax ester metabolism profile of *S. purpurea* and increased its adaptability to an extreme drought environment.

## Introduction

Drought is a main limiting factor in species distribution (Engelbrecht *et al.*, 2007). Currently, progress is being made in revealing plant adaptive response mechanisms to water stress by studying micro-mechanisms, biochemistry, physiology, and ecology, and in elucidating the intensity of water stress and the correlation between water stress intensity and plant responses and adaptations. Plant cuticular waxes play important roles in resistance to drought and other adverse stresses (Zhou *et al.*, 2018; Patwari *et al.*, 2019). The synthesis of plant cuticular waxes is a complex process, which includes three main important stages (Samuels *et al.*, 2008; Patwari *et al.*, 2019). First, C16 and C18 fatty acids are synthesized *de novo* by fatty acid synthases. In the second stage, very-long-chain fatty acids with C20–C34 chains are produced using C16 or C18 fatty acids and malonyl-CoA as substrates. They are catalyzed by a fatty acid elongase complex containing a β-ketoacyl-CoA synthase, a β-ketoacyl-CoA reductase, a β-hydroxyacyl-CoA dehydratase, and an enoyl-CoA reductase (Huanquan *et al.*, 2005; Kunst and Samuels, 2009). In the third stage, very-long-chain fatty acids are either modified into aldehydes, alkanes, and ketones by fatty acyl-CoA reductases (CER1 and CER3) and a midchain alkane hydroxylase in the alkane-forming pathway, or modified into primary alcohols and wax esters by fatty acyl-CoA reductase (CER4) and the wax ester synthase/diacylglycerol acyltransferase (WSD) in the alcohol-forming pathway (Rowland *et al.*, 2006; Li *et al.*, 2008; Amélie *et al.*, 2012).

Drought stress up-regulates wax biosynthetic genes to stimulate cuticular wax accumulation (Zhou *et al.*, 2018). Furthermore, mutations in genes related to wax biosynthesis show altered wax accumulations that can lead to decreases in drought resistance capabilities. For example, the *lacs1 lacs2* double mutant of *Arabidopsis thaliana* has greater leaf cuticular permeability and drought stress susceptibility than wild-type plants (Weng *et al.*, 2010). A *glossy 1* (Arabidopsis CER1 homologous gene) mutant in rice (*Oryza sativa*) shows decreased cuticular wax depositions and an increased sensitivity to drought stress compared with wild-type rice. However, the overexpression of *glossy 1* (OsGL1-2) provides a high resistance level against drought stress, compared with the wild type, owing to the presence of increased cuticular wax levels (Islam *et al.*, 2009). In addition, the wax ester level increases in *A. thaliana* in response to a water deficiency (Patwari *et al.*, 2019). WSD1, as a member of the wax ester synthase/diacylglycerol acyltransferase (WS/DGAT) family in plants, plays a key role in wax ester synthesis, and a reduction in wax esters may decrease drought stress tolerance. Recombinant expression analyses of WSD1 in yeast and *Escherichia coli* revealed predominant wax synthase functions, and showed that it could synthesize different wax ester products, including C34, C40, and C44, using different substrate combinations of primary alcohols and primary acids (Li *et al.*, 2008). The substrate selectivity of WS/DGAT enzymes from *Marinobacter aquaeolei* can be altered through mutations in specific residues, resulting in the synthesis of different wax ester components (Barney et al., 2013, 2015; Petronikolou *et al.*, 2018).

As the highest plateau in the world, with a mean elevation of >4000 m, the Qinghai–Tibet Plateau (QTP), covers ~2.5 million km^2^ and contains enormous glaciers, huge alpine lakes, and impressive waterfalls (Adrien *et al.*, 2015). In northern Tibet, a strong ecological gradient in precipitation from east to west (20 mm to 600 mm) is attributed to the southwestern monsoon wind system (Caiming *et al.* 2008). In this region, the tussock grass steppe is the most typical land form, which supports species of the genus *Stipa* L. from the Poaceae family. Among these species, *Stipa capillacea* is mainly distributed in the relatively abundant alpine meadows in the eastern part of northern Tibet, while *Stipa purpurea* is mainly distributed in the relatively dry areas. The two closely related *Stipa* species form a geographically related distribution from east to west, providing a rare opportunity to understand the physiological and molecular strategies required for plant adaptation to harsh environmental conditions. Moreover, there is little knowledge regarding differences in adapting to drought conditions among related species growing in different rainfall regions.

Species from these unique environments on the QTP have undergone genetic divergence under long-term selective pressure to increase their adaptability (Zhang *et al.*, 2019). Adaptive protein evolution among species can be described using the signature of positive selection to calculate the ratio of non-synonymous (dN) to synonymous (dS) substitutions (Zhang *et al.*, 2005). For example, in Tibetans, *Egl nine homolog 1*, *peroxisome proliferator-activated receptor-a*, and endothelial *PAS domain-containing protein 1* genes contain signatures of positive selection and are involved in the hypoxia-inducible factor-1 signaling pathway responsible for the acquisition of molecular adaptations to high altitude (Simonson *et al.*, 2010). In addition to hypoxia response genes, energy metabolism- and skeletal development-related genes exhibit positive selection in vertebrates, such as yak, Tibetan wild boar, and Tibetan mastiffs (Simonson, 2015). In contrast, few studies have applied next-generation sequencing to analyze the effects of positively selected genes on the adaptation of wild plants to the QTP. Recently, positively selected genes in *Crucihimalaya himalaica* were identified based on transcriptome and whole-genome analyses, and the functions of these genes were predicted to increase our understanding of plant adaptive mechanisms to extreme environmental conditions (Qiao *et al.*, 2016; Zhang *et al.*, 2019).

A protein’s function can be optimized through positive selection. The interaction intensity between *A. thaliana* AtPOT1a and AtCTC1 is enhanced by three positive selection sites in AtPOT1a, suggesting that an ancestral function was reinforced, rather than neofunctionalization (Beilstein *et al.*, 2015). In this study, we investigated the drought resistance capabilities of *S. capillacea* and *S. purpurea* from different locations in an ecological drought gradient and investigated how transcriptional divergence correlated with their unique phenotypes and acclimation to their local environments. Our results indicate that the *WSD1* genes of *S. purpurea* which correlated with synthesis of cuticular waxes contain positive selection sites. Positive selection sites may result in changes to the wax ester profile, which may be due to the change of substrate selection by enzymes in the wax ester synthetic pathway, thereby contributing to the plant’s adaptation to drought stress conditions. Our results increase our understanding of how transcriptome divergence correlates with adaptive diversity in wild plants on the QTP and also support a new model in which positively selected sites may be important for the optimization of protein functions.

## Materials and methods

### Seed collection and seedling breeding

**Fig. 1.**
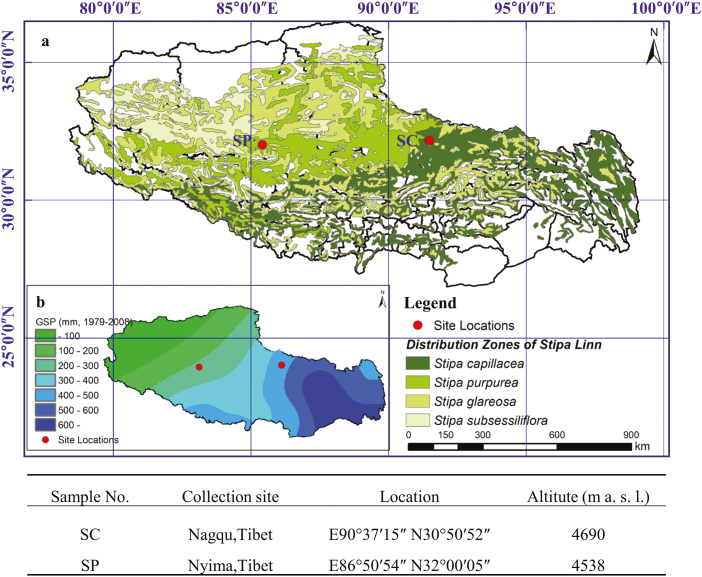
Plant materials and relevant environmental conditions. (a) The collection site locations, and detailed information on the collected seeds. (b) The annual average precipitation from 1979 to 2008 on the QTP. SC, *S. capillacea*; SP, *S. purpurea.*

Mature seeds of *S. capillacea* and *S. purpurea* were collected from the QTP (Fig. 1). After being brought to the laboratory, the seeds (50 per pot) were sown in flower pots (diameter=9.4 cm, height=8 cm) containing equal amounts of humus soil located in a glasshouse under controlled conditions (12 h light/12 h dark cycle; 28 °C day/25 °C night cycle, 200 mmol photons m^−2^ s^−1^ light intensity, relative humidity of 75–80%) until they germinated. The samples were first sufficiently watered, and the soil water content was calculated by dividing the soil water content by soil dry weight. Every day at 16.00 h, each sample was watered to maintain a constant soil moisture content.

### Drought stress

When the seedlings reached the trefoil stage (after ~3 weeks of growth), two stages of continuous drought stress were instituted by withholding watering. Water was withheld from half of the seedlings of each *Stipa* species for 7 d, and then they were re-watered for another 7 d. Water was withheld from the remaining seedlings for 11 d (one plant appeared to die), and then they were re-watered for another 7 d. Samples from plants subjected to drought stress conditions for 0, 7, or 11 d and rehydration for 7 d or 11 d were collected for subsequent measurements. The samples from plants grown under drought conditions for 0 and 7 d and rehydrated for 7 d were collected, frozen in liquid nitrogen, and kept at −80 °C prior to use for RNA isolation. Three replicates were collected and analyzed per treatment.

### Analysis of leaf water content and chlorophyll fluorescence

The leaf relative water contents (RWCs) of the third mature leaves of the two *Stipa* species were calculated as: RWC (%)=(FW−DW]/(Turgid weight−DW)×100. To determine the fresh weights, leaves were weighed immediately after collection. The turgid weight was obtained from leaves kept in distilled water in darkness at 4 °C to minimize respiration losses until their moisture contents reached a constant weight. The dry weights of leaves were then determined after 48 h in an air oven at 70 °C. Chlorophyll fluorescence was measured using a pulse-amplitude modulation chlorophyll fluorometer (Heinz Walz GmbH, Effeltrich, Germany). Briefly, seedlings of the two *Stipa* species were dark adapted for 30 min to measure the maximum quantum yield of PSII [variable fluorescence (*F*_v_)/maximum fluorescence (*F*_m_)]. The *F*_m_ was recorded after a 0.8 s pulsed light at 8000 μmol s^−1^ m^−2^, and the minimal fluorescence was recorded during the weak measuring pulses.

### PacBio Iso-Seq library preparation and sequencing

Total RNA extraction was performed using RNAiso Plus (Qiagen, Valencia, CA, USA) and the quality was assessed using an Agilent 2100 Bioanalyzer. To obtain full-length transcriptome information, poly(A)^+^ RNA was purified, and was reverse transcribed using the SMARTer PCR cDNA Synthesis Kit (Clontech, Mountain View, CA, USA). Then, cDNA fractions were selected using BluePippin (Sage Science, Beverly, MA, USA). PCR amplification of cDNA fractions was performed using 12 cycles, and products were made into SMRTbell Template libraries according to the Iso-Seq protocol. cDNA libraries were selected for the following bins: 1–2 kb, 2–3 kb, and 3–6 kb, and sequenced on a PacBio RS II instrument.

### Illumina RNA-seq sequencing

Total RNAs were isolated from plants during various stages of the drought treatments, and poly(A)^+^ RNAs were purified and interrupted to form short fragments by adding fragmentation buffer. Next, a random hexamer primer and Superscript II (Invitrogen, Carlsbad, CA, USA) was used to synthesize the first-strand cDNA using these short fragments as templates, and then the second-strand cDNA was synthesized using RNase H and DNA polymerase I. Fragments were retrieved and connected using sequencing adaptors. Next, fragments were selected after separation by agarose gel electrophoresis, and then suitable fragments were amplified as templates. The libraries were paired-end (100 bp) sequenced using an Illumina HiSeq 2000 (San Diego, CA, USA). Clean reads were obtained by removing adaptors, and unknown (‘N’>5%) and low-quality bases (scores <20) reads, from the raw reads.

### Analysis of the full-length transcriptome

According to the PacBio protocol, raw polymerase reads were processed into error-corrected reads of inserts (ROIs) using the Iso-Seq pipeline with the criteria minFullPass=0 and minPredictedAccuracy=0.80. Next, full-length, non-chimeric transcripts were identified by searching for poly(A) tail signals and the 5' and 3' cDNA primers in the ROIs. Iterative clustering for error correction was used to obtain consensus isoforms, and these full-length consensus sequences were polished using Quiver, producing high-quality and polished full-length consensus sequences. Finally, these polished consensus sequences were further subjected to correction and redundancy removal compared with Illumina short reads using the LoRDEC tool (Leena and Eric, 2014) and CD-Hit (identity >0.99) (Fu *et al.*, 2012), respectively.

### Bioinformatic and differential expression analysis

For the comprehensive functional annotation, the full-length transcriptome was aligned against protein databases, including the NCBI non-redundant protein (Nr), Swiss-Prot, KEGG, and COG (e-value ≤10^−5^). When different databases produced conflicting results, they were prioritized as: Nr>Swiss-Prot>KEGG>COG (Ye *et al.*, 2006). For the differential expression analysis, full-length isoform transcripts were used as reference sequences, and the clean reads obtained by Illumina RNA-Seq were mapped independently to the full-length isoform transcripts using the TopHat 2.0 program (Trapnell *et al.*, 2012). For the differential expression analysis between the two *Stipa* species, the fold changes were screened using EdgeR (Robinson *et al.* 2010) in R (R Core Team, 2013) using the log2 ratio of FPKM (fragments per kilobase of transcript per million mapped reads). *P*-values were adjusted using the false discovery rate (FDR) in multiple tests and analyses. We used the criteria of FDR≤0.01 and the |log2(fold change)|≥2 as thresholds to judge the significance of gene expression differences.

### Analysis by quantitative real-time PCR (qRT-PCR)

To examine the expression of the investigated genes, qRT-PCR was performed as previously described (Bennett *et al.*, 2015) using the following conditions: 40 cycles at 95 °C for 5 s, 60 °C for 30 s, and 72 °C for 15 s. *S. purpurea Actin 1* (GenBank: KM216249) was used to normalize the samples from the two *Stipa* species because they shared the same nucleic acid sequence. The primer sequences are shown in Supplementary Table S1 at *JXB* online).

### Histology

SEM was performed to evaluate cuticular wax crystals. The third mature leaves from the same general locations on plants were sliced into 0.5 cm sections, fixed in 1% osmium tetroxide vapor for 24 h, and allowed to air dry for 24 h. Then, specimens were coated with platinum particles and observed using an FEI QUANTA 200 3D equipped with a large-field secondary electron detector and operated at 20 kV. Stomatal density was determined from *S. capillacea* and *S. purpurea* plants using the samples for SEM. The number of stomata was counted in the abaxial surface of leaves, and the areas were calculated according to the scale of the SEM image. Stomatal density was calculated as stomatal number/area of leaves. In addition, a TEM analysis was performed as described by Yang *et al.* (2015). Fresh leaves were pre-fixed for 12 h in 2% (v/v) glutaraldehyde in PEMT buffer (pH 7.2) at 4 °C. After rinsing in phosphate buffer (pH 7.2), post-fixation was performed in 2% (v/v) osmium tetroxide for 2 h. Then, the samples were dehydrated first through a gradient series of ethanol concentrations (30, 50, 70, 85, 95, and 100%), infiltrated with acetone, and embedded in low-viscosity Spurr’s resin (Electron Microscopy Sciences). The samples were cut 60 nm thick, stained for 1 min with 2.5% uranyl acetate, and viewed using a JEM-1230 transmission electron microscope (JEOL, Tokyo, Japan).

### Cuticular wax analysis by GC-MS

Cuticular waxes were extracted from 1 g of leaves. Each sample was dipped in 10 ml of chloroform for 30 s, and then 2 μg of C15:0 was included as an internal standard (Li *et al.*, 2008). The solvent was evaporated under nitrogen gas at 40 °C and the wax mixtures were treated with 300 μl of *n*-hexane to dissolve the sample. After adding 2 ml of 5% methanol sulfuric acid, the mixture was heated in a water bath at 85 ° C for 1.5 h. Then, 750 μl of 1% KCl was added to the sample, washed three times, and the supernatant was collected and dried under vacuum. The sample was dissolved with 300 μl of *n*-hexane, and was analyzed by GC-MS (Agilent 7890A/5975C). A 1 μl aliquot of solution was injected using an HP 7683 auto sampler (Agilent) and introduced in split mode (1:20) for the quantitative element analysis, applying the following parameters: oven 2 min at 50 °C, raised by 40 °C min^−1^ to 200 °C, injection at 220 °C for 2 min, an increase of 3 °C min^−1^ to 320 °C, maintained for 30 min at 320 °C, and He carrier gas inlet pressure was programmed for a constant flow of 1.4 ml min^−1^. Products were identified using the National Institute of Standards and Technology (NIST) search engine, version 2.0f (Agilent). The quantitative analyses of wax mixtures were performed by comparing GC-FID peak areas against the internal standard and dividing by the injection volume determined for the corresponding sample.

### The rate of water loss and chlorophyll leaching analysis

The rate of water loss from detached leaves of *S. capillacea* and *S. purpurea* was determined according to Chen *et al.* (2003). The third fully expanded leaves were detached and fully soaked in distilled water for 60 min in darkness. After removing excess water in complete darkness, weights were determined gravimetrically at 30, 60, 90, 120, 150, and 180 min using a microbalance under controlled conditions (28 °C, relative humidity of 30–40%). The rate of water loss was calculated based on the weight of water lost divided by the initial leaf weight. The chlorophyll leaching experiment was performed as previously described, with minor modifications (Mao *et al.*, 2012). In brief, the leaves from *S. capillacea* and *S. purpurea* were weighed, cut into ~3 cm lengths, and extracted in 15 ml of 80% (v/v) ethanol with gentle agitation in the dark. A 200 μl aliquot of the supernatant was removed at 30, 60, 90, 120, 150, and 180 min, and the absorbance of the supernatant at 664 nm and 647 nm was determined. The chlorophyll concentration was calculated according to the formula: Ci=7.93×*A*_664_+19.53×*A*_647_.

### *WSD1* genes identified and positive selection analysis

Publicly available coding and predicted protein sequences were downloaded from several databases, including Phytozome (v13) databases for *Brachypodium distachyon* (v3.1), *Oryza sativa* (v7.0), *Zea mays* (Ensembl-18), and *Setaria italica* (v2.2), Ensembl Plants for *Aegilops tauschii* (v4), and Plant Genome and Systems Biology for *Hordeum vulgare.* WSD1 sequences of several gramineous plants were obtained according to Rosli *et al.* (2018), and used as queries to search against predicted protein sequences of *S. capillacea*, *S. purpurea*, and the above genomes. All the WSD1 protein sequences containing a WES_acyltransf domain (PF03007), DUF1298 (PF06974). and the HHXXXDG motif were extracted as candidates. The *WSD1* nucleotide sequences were aligned using the MAFFT (version 7) program, and phylogenetic trees were constructed using PhyML 3.0 software with the GTR+GAMMA model, the maximum likelihood criteria. and 1000 bootstrap test replicates (Guindon *et al.*, 2010). For positive selection assessment, the CODEML program in the PAML 4.8 software package was used based on a site-specific model (Yang, 2007). Models M1a (nearly neutral), M2a (positive selection), M7 (beta), and M8 (beta+ω) were all used in this analysis. The significant likelihood ratio tests compared M1a with M2a, and M7 with M8, to determine dN and dS nucleotide divergences in coding regions. An empirical Bayes approach was used to calculate the posterior probability that each site belongs to the positive selection class in each model.

### Analysis of heterologous expression of *WSD1* in yeast and wax esters

The full-length cDNAs of *WSD1* were independently isolated from *S. purpurea* (GeneBank ID: MK994184) and *S. capillacea* (GenBank ID: MK994185). *SpWSD1* and *ScWSD1* were independently subcloned into the pESC-URA vector (Invitrogen) behind the Gal-inducible GAL1 promoter. To mutate *SpWSD1* and *ScWSD1*, the two mutational plasmids, *SpWSD1*-m (T239S) and *ScWSD1*-m (S239T), were constructed using the Fast Mutagenesis System (TransGen Biotech, Beijing, China) according to the manufacturer’s instructions. Primers are listed in Supplementary Table S2. The pYES-URA-*SpWSD1*, -*ScWSD1*, -*SpWSD1*-m, and -*ScWSD1*-m vectors, as well as an empty control vector, were independently transformed into the mutant *S. cerevisiae* strain H1246, which is defective in storage lipid accumulation according to Sandager *et al.* (2002).

Total lipids were extracted according to Li *et al.* (2008). Transgenic yeast cells were cultivated in synthetic minimal dropout medium (50 ml) without uracil and containing 2% (w/v) Gal for 48 h at 30 °C. Primary alcohols (C18) and palmitic acid (16:0) were added to the culture medium to a final concentration of 0.1% (w/v). Then, yeast cells were collected, washed twice with water, and resuspended in 2 ml of methanol. Glass beads (0.5 mm) were added and the suspension was vigorously vortexed. After 5 min, lipids were extracted by adding 4 ml of chloroform and 1 ml of 0.9% NaCl. The sample was mixed by vortexing, and the chloroform fractions were transferred, dried under nitrogen, and resuspended in 20 μl of chloroform. Wax esters were analyzed using GC-MS as described above for the plant wax analysis.

## Results

### Different physiological responses in two *Stipa* species under dought stress

**Fig. 2.**
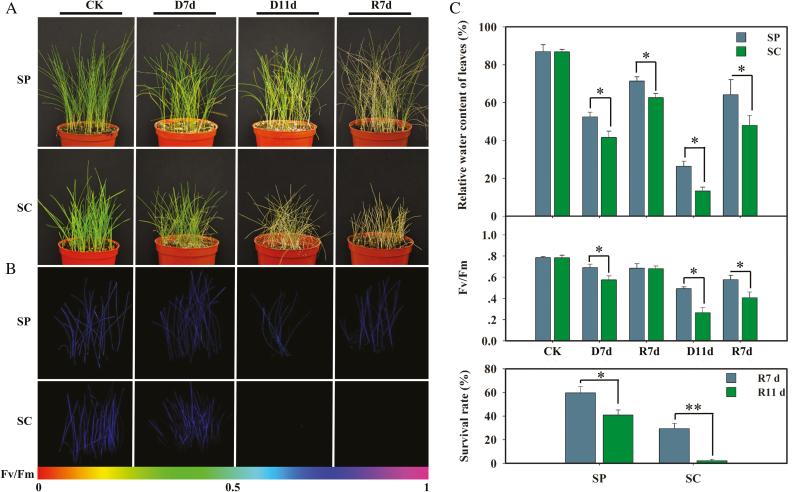
*Stipa capillacea* and *S. purpurea* phenotypes and physiological responses under different water conditions. (a) Leaf phenotypes of *S. capillacea* and *S. purpurea* following exposure to drought stress. (b) *F*_v_/*F*_m_ images were captured using a PAM chlorophyll fluorometer. The code depicted at the bottom of the image ranges from 0 to 1.0. (c) Variations in the relative water contents of leaves, *F*_v_/*F*_m_ values, and survival rates of *S. capillacea* and *S. purpurea* in response to drought stresses. Data (mean values ±SEs) were obtained from three replicate experiments (Student’s *t*-test, *P<0.05, **P<0.01).

To compare the effects of various drought levels on the viabilities of *S. capillacea* and *S. purpurea*, the water contents of leaves were determined under drought and re-watering conditions. As shown in Fig. 2a, when treated with drought for 7 d, the leaves of both species of *Stipa* gradually began to change from green to yellow, with no significant difference in morphology between the two species. However, after 11 d drought treatment, significant phenotypic differences were observed between the two species; some leaves of *S. purpurea* had already dried out, with a more pronounced change in *S. capillacea*. The leaf RWC of *S. capillacea* after 11 d of drought decreased to 11.90% compared with well-watered conditions, which was significantly lower than the RWC of *S. purpurea* (25.31%) (Fig. 2b). Meanwhile, we compared the maximum photochemical efficiencies of PSII between the two *Stipa* species, as reflected by *F*_v_/*F*_m_ values. When treated with drought for 11 d, the *F*_v_/*F*_m_ of *S. capillacea* decreased by 0.38, whereas that of *S. purpurea* decreased from 0.79 to 0.57. There was a significant difference between the values of the two *Stipa* species (Fig. 2c). When re-watered for 11 d, only a few *S. capillacea* (1.93%) were alive, while ~40% of *S. purpurea* were still alive (Fig. 2d). These morphological and physiological response differences directly demonstrated that the drought resistance of *S. purpurea* was stronger than that of *S. capillacea*.

### Transcriptome analysis of *Stip*a species using PacBio Iso-Seq

To obtain the full-length transcriptomes of the *Stipa* species, PacBio Iso-Seq was performed using leaf tissues from plants receiving different treatments. A total of 337 754 and 335 190 ROIs were generated using five SMRT cells each from *S. purpurea* and *S. capillacea* (Supplementary Table S3). Applying the standard Iso-Seq classification and clustering protocol, we produced 54 509 polished high-quality and 14 039 low-quality transcripts for *S. purpurea* leaf tissue, and 49 908 polished high-quality and 12 138 low-quality transcripts for *S. capillacea* leaf tissue (Table 1). After removal of redundant transcripts, 59 608 and 54 729 full-length consensus transcripts for *S. purpurea* and *S*. *capillacea* leaf tissues, respectively, remained (Table 1). These full-length consensus transcripts were functionally annotated based on the proteins in the Nr, Swiss-Prot, KEGG, GO, KOG, Pfam EggNOG, and COG databases with the highest sequence similarity levels. In total, 57 835 transcripts (97.03%) of *S. purpurea* and 53 176 transcripts (97.17%) of *S. capillacea* were annotated in the databases (Table 2). Based on Nr annotations, ~50% of the full-length transcripts from each *Stipa* species had top matches with *B. distachyon* sequences (Supplementary Fig. S1).

**Table 1.** Summary of the consensus transcripts after using the standard PacBio Iso-Seq bioinformatics pipeline

Samples	*S. purpurea*				*S. capillacea*			
Size	0–2 kb	2–3 kb	3–6 kb	>6 kb	0–2 kb	2–3 kb	3–6 kb	>6 kb
Number of polished high-quality isoforms	25 698	18 522	10 289	16	19 523	19 771	10 614	21
Number of polished low-quality isoforms	4364	4202	5473	702	2772	4550	4916	919
Average consensus isoforms read length	1428	2337	3586	8940	1327	2,359	3476	9130
Number of corrected transcripts	59 608				54 729			

**Table 2.** Numbers of annotated transcripts from *S. purpurea* and *S. capillacea*

Name	GO	KEGG	KOG	Pfam	Swissprot	COG	EggNOG	Nr	All
Annotated number of *S. purpurea*	50 729	25 748	37 221	48 353	43 276	25 436	57 521	57 696	57 835
(%)	85.10	43.20	62.44	81.12	72.60	42.67	96.50	96.79	97.03
Annotated number of *S. capillacea*	46 877	24 276	34 183	44 214	39 851	23 741	52 913	53 116	53 176
(%)	85.65	44.36	62.46	80.79	72.82	43.38	96.68	97.05	97.16

### Transcriptome changes in two *Stipa* species under dought stress

**Fig. 3.**
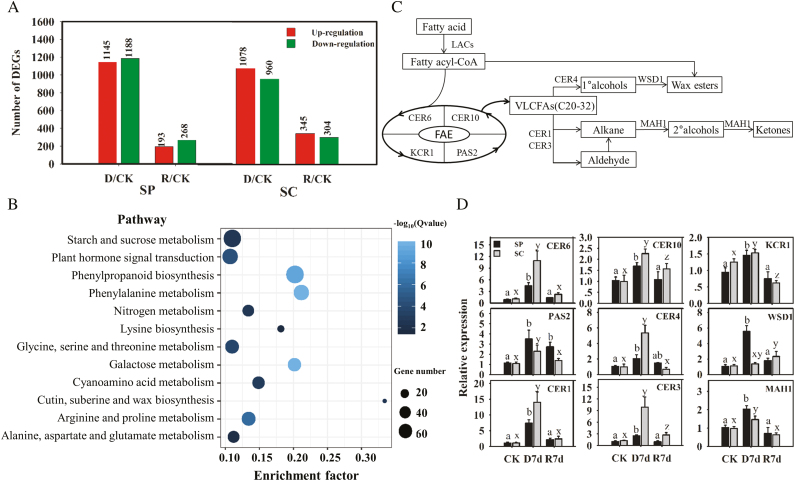
The roles of cuticular wax biosynthetic genes of *S. capillacea* and *S. purpurea* in response to drought conditions. (a) Numbers of differentially expressed genes that were significantly up- or down-regulated at the transcriptional level in *S. capillacea* and *S. purpurea* under drought conditions. (b) KEGG pathway analysis of differentially expressed genes of *S. purpurea* during drought compared with those of the control. The enrichment factor indicates the ratio between the number of differentially expressed genes and all the annotated genes in this pathway. The value of the enrichment factor represents the degree of enrichment. The 12 most significantly enriched pathways are shown. (c) Schematic diagram of the cuticular wax biosynthetic pathway. (d) Relative expression levels of wax biosynthetic genes. The bars indicate the means ±SEs of three replicate experiments (*P*<0.05; Tukey’s test).

To further investigate the gene expression profiles of *Stipa* species’ responses to drought stress, their clean RNA-seq reads were mapped independently to the reference transcriptome (Supplementary Tables S4, S5), which was obtained using PacBio Iso-Seq because *S. purpurea* is not a ‘model’ plant and, therefore, no public genomic information was available. Gene expression levels were calculated using the FPKM method, and genes differentially expressed between different treatments and their controls were determined according to |log2(fold change)|≥2 and FDR values <0.01 (Supplementary Fig. S2). Compared with the control level (0 d), 1145 and 1188 transcripts from *S. purpurea* showed up- and down-regulated expression levels, respectively, during the drought treatment (Fig. 3a). After rewatering, 193 up- and 268 down-regulated transcripts were differentially expressed in *S. purpurea* (Fig. 3a). In addition, 2038 transcripts, 1078 up-regulated and 960 down-regulated, in *S. capillacea* were differentially expressed during the drought treatment. After rewatering, there were 649 transcripts, 345 up-regulated and 304 down-regulated, in *S. capillacea* that showed differential expression levels (Fig. 3a). Thus, there were greater numbers of genes exhibiting down-regulation in the two *Stipa* species during the recovery than during the drought treatment. Furthermore, the differentially expressed genes of the two *Stipa* species during the drought treatment were categorized using a KEGG pathway analysis. The cutin, suberin, and wax biosynthetic pathways had the greatest enrichment factor, indicating that the metabolic pathways involving these genes were important for drought adaptation in *S. purpurea* (Fig. 3b, c; Supplementary Fig. S3).

### Expression of wax biosynthesis genes during dought stress

To further determine the expression levels of genes involved in wax synthesis in the two *Stipa* species during the drought treatments, nine differentially expressed genes were selected and their expression profiles were assessed by qRT-PCR. The nine genes were significantly up-regulated in both *Stipa* species during the drought treatment, which was consistent with the transcriptome data (Fig. 3d; Supplementary Fig. S4). The highest levels of *CER1* were observed after 7 d of drought treatment, which were increased by ~7.5-fold in *S. purpurea* and 14-fold in *S. capillacea*, respectively. In addition, homologous genes from the two species also showed significant differences in relative expression levels. Compared with *S. purpurea*, the *CER1*, *CER3*, *CER4*, and *CER6* genes of *S. capillacea* were up-regulated significantly more highly than those of *S. purpurea* after drought treatments (Fig. 3d). Conversely, the increased expression levels of *WSD1* and *MAH1* from *S. purpurea* were higher than the expression level of *WSD1* from *S. capillacea* after drought treatments. Taken together, these results indicate that differences in homologous gene expression in the waxy synthetic pathway of *S. purpurea* and *S. capillacea* may lead to differences in drought resistance between the two species.

### Wax content changes in two *Stipa* species under dought stress

**Fig. 4.**
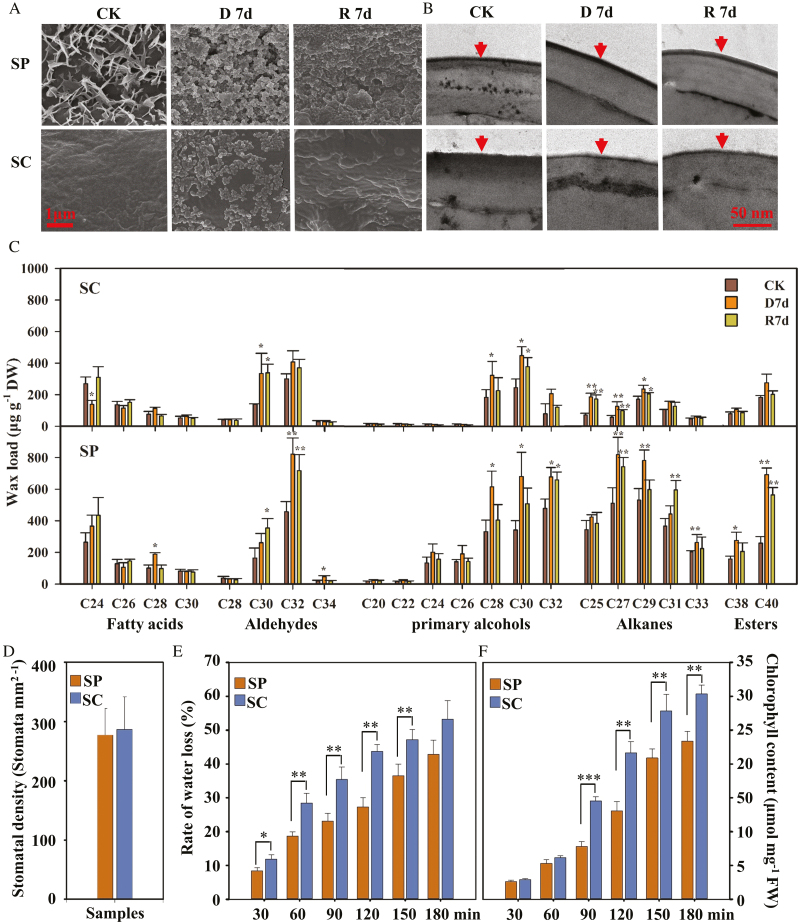
Leaf cuticular waxes and the rate of water loss of two closely related *Stipa* species after exposure to drought. (a) SEM images of cuticular wax crystals on leaves of *S. capillacea* (SC) and *S. purpurea* (SP) under drought conditions. Scale bar=1 μm. (b) TEM images of cuticle layers in leaf epidermal cells of SC and SP under drought conditions. The cuticle is indicated by an arrow. Scale bars=50 nm. (c) Cuticular wax amounts and composition on leaves of SC and SP under drought conditions. Cuticular waxes were analyzed by GC. (d) The stomatal density was calculated as described in the Materials and methods. Comparison of water loss rates (e) and chlorophyll leaching (f) of the detached leaves of SC and SP in a dark environment. The vertical bars represent the means ±SEs of three replicate experiments; significant differences at *P<0.05, **P<0.01, and ***P<0.001.

To analyze the effects of cuticular waxes on the responses of the two *Stipa* species during drought stress, we determined the deposition levels of cuticular wax crystals on the leaf surfaces of both species using SEM and TEM. Under normal conditions, there were more cuticular wax crystals on the leaf surface of *S. purpurea* than on *S. capillacea* (Fig. 4a, b). When they were exposed to dehydration and rehydration treatments, we observed that the cuticular wax crystals on the leaf surfaces of *S. purpurea* were more abundant in their distribution compared with those of *S. capillacea* (Fig. 4a, b). We subsequently determined the chemical composition of the cuticular waxes on *S. purpurea* and *S. capillacea* leaves using GC-MS. Under normal conditions, *S. purpurea* had greater C24, C26, C28, C30, and C32 primary alcohol, as well as alkane, and C38 and C40 ester contents, compared with *S. capillacea* (Fig. 4c). In particular, the amounts of C30 aldehyde, C27 and C29 alkanes, and C28 and C30 primary alcohols were significantly increased in the two *Stipa* species in response to drought stress. Additionally, compared with *S. capillacea*, the amounts of some compounds, including C32 aldehyde, C31 and C33 alkanes, C32 primary alcohols, and C38 and C40 esters, were significantly increased in *S. purpurea* leaves relative to the controls (Fig. 4c). The lack of detection of cuticular wax precursors such as C16 and C18 fatty acids may be due to the fact that samples of the entire leaves were shaken in chloroform for 30 s.

Cuticular waxes and stomata can control leaf water loss in plant survival under severe water deficit. In order to further detect the effect of cuticular waxes on leaf water loss of the two *Stipa* species, we first measured the stomatal density based on leaf samples by SEM. The results showed that there was no significant difference in stomatal density between the two *Stipa* species (Fig. 4d). Subsequently, the water loss rate from detached leaves was checked after stomatal closure caused by a dark environment for 1 h (Supplementary Fig. S5). As shown in Fig. 4e, water was lost more quickly from the *S. capillacea* than from the *S. purpurea* leaves at all the points examined. In particular, water loss rates of detached leaves in *S. capillacea* were >1.60-fold higher compared with those of *S. purpurea* at the 120 min point. In addition, epidermal permeability was also measured using a chlorophyll leaching assay. The results showed that chlorophyll leaching was greater in *S. capillacea* leaves than in *S. purpurea* after 90 min of processing (Fig. 4f). These results indicate that *S. capillacea* were more sensitive to water deficit than *S. purpurea*, implying that the cuticular waxes may be involved in *S. purpurea* drought protection.

### *WSD1* gene positive selection analysis

**Fig. 5.**
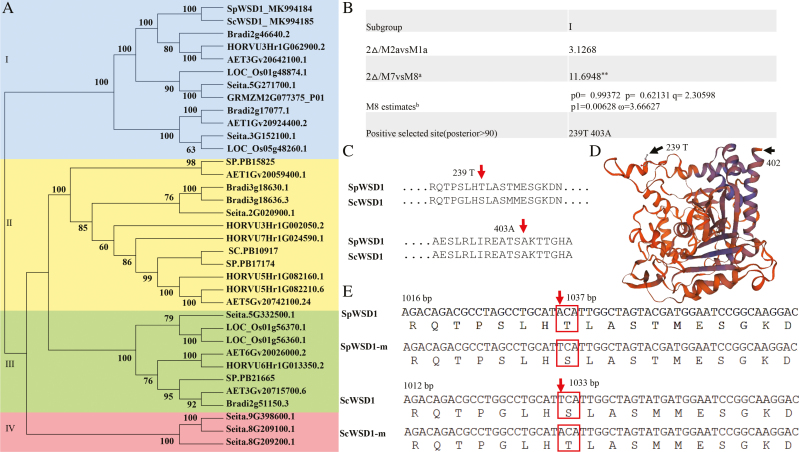
The identified *WSD1* genes and a positive selection analysis in eight representative plants. (a) Phylogenetic trees were constructed using PhyML 3.0 software with the GTR+GAMMA model, the maximum likelihood criteria, and 1000 bootstrap test replicates. The four subgroups, I–IV, are shown. SpWSD1 GenBank accession no. MK994184, ScWSD1 GenBank accession no. MK994185. (b) Likelihood values and parameter estimates for the WSD1 genes of subgroup I. Positive selection site tests of eight related species using the site-specific model in the PAML 4 package, and the positive selection sites of subgroup I predicted by the M8 model. (c) The positive selection sites are labeled for SpWSD1 and ScWSD1. (d) Predicted protein structures of SpWSD1. The crystal structures were predicted using SWISS-MODEL (https://swissmodel.expasy.org/). 259T was labeled on the predicted protein crystal structure, 403A was not labeled due to the incomplete C-terminal fragment of the predicted structure. (e) Diagram of the site-directed mutagenesis for SpWSD1 and ScWSD1.

Genomes of six relatives, *B. distachyon*, *O. sativa*, *Z. mays*, *S. italica*, *A. tauschii*, and *H. vulgare*, and predicted protein sequences of *S. capillacea* and *S. purpurea* were selected to identify *WSD1* gene family members. Previous genome-wide analyses identified five, four, and one *WSD1* gene in *B. distachyon*, *O. sativa*, and *Z. mays*, respectively (Rosli *et al.*, 2018). In this study, we identified seven, six, and seven *WSD1* genes in *H. vulgare*, *A. tauschii*, and *S. italica*, respectively, and four and two *WSD1* genes were identified using the full-length transcriptomes of *S. purpurea* and *S. capillacea*, respectively (Fig. 5a; Supplementary Table S6). A phylogenetic analysis suggested that four groups were generated in the eight plant species (Fig. 5a), subgroups 1, 2, 3, and 4. Amino acids in a protein sequence are expected to be under different selective pressure and to have different underlying dN/dS ratios. To test for positive selection at individual amino acid codons, the site-specific models, M1a, M2a, M7, and M8, implemented using the CODEML program of the PAML v4.8 package were applied to each group. The likelihood ratio tests (2Δ|lnL) were constructed through comparisons of two pairs of site models, M1a and M2a, and M7 and M8 (Supplementary Table S7). The WSD1 sequences within each of the subgroups are under strong purifying selection pressure, which may have acted on only a few sites during the evolutionary process (Supplementary Table S7). The detailed distribution of the positive selection sites in subgroup 1 sequences as predicted by the M8 model is shown in Fig. 5b. Two amino acid sites (239T and 403A) were under positive selection (Fig. 5c; Supplementary Table S7). Interestingly, SpWSD1 from *S. purpurea* showed a difference at the 239T site compared with related species (Fig. 5c). In addition, a threonine residue at position 239 was identified as a positive selection site in SpWSD1 (cut-off=90%), and was distributed in α-helices (Fig. 5d), whereas there was a serine at this site in ScWSD1 (Fig. 5c, e). Thus, SpWSD1 might play an important role in wax synthesis that enhances the drought resistance of *S. purpurea*, and this site may have a specific function.

### Functional analysis of WSD1 positive selection sites using heterologous expression in yeast

**Fig. 6.**
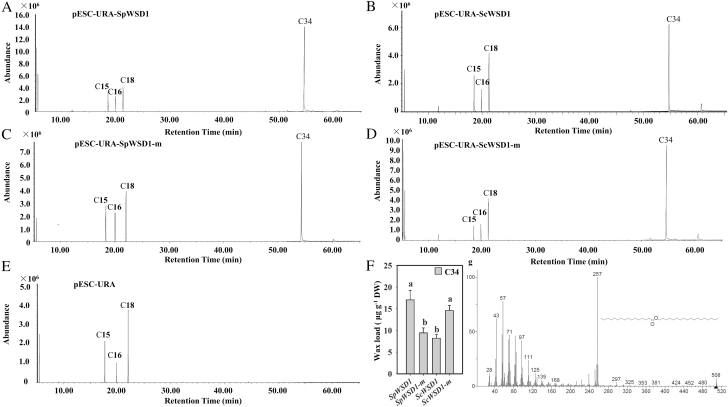
Wax ester analysis using WSD1 heterologously expressed in yeast. The contents of wax esters were analyzed by GC. Primary alcohols (C18) and palmitic acid (16:0) were used as substrates to feed yeast, and C15:0 was included as an internal standard. (a–e) Recombinant yeast H1246 expressing SpWSD1 (a), ScWSD1 (b), SpWSD1-m (c), ScWSD1-m (d), and empty vector (e). (f) Amounts of wax esters. (g) Mass spectrum of C34 wax esters (molecular ion *m/z* 508). Data (mean values ±SEs) were obtained from three replicate experiments (Student’s *t*-test, *P<0.05, **P<0.01).

Because the T239 residue was identified as a positive selection site in SpWSD1, and WSD1 contributes to the wax ester synthesis of leaves and stems (Li *et al.*, 2008; Patwari *et al.*, 2019), we investigated the effects of positive selection sites on WSD1 activity. The mutant T239S, named *SpWSD1-*m, was constructed at the T239S site of SpWSD1, and the mutant S239T, named *ScWSD1*-m, was constructed at the S239 site of ScWSD1 (Fig. 5d; Supplementary Table S7). Four *WSD1* cDNA fragments were introduced into the yeast mutant H1246 that is deficient in storage lipid biosynthesis (Sandager *et al.* 2002). The wax esters produced revealed that *SpWSD1* and *ScWSD1* are members of the bifunctional *WSD* gene family, and all of the recombinant yeast were involved in wax formation (Fig. 6a–g). Recombinant yeast harboring SpWSD1-m with the mutant site S239T could synthesize fewer C34 esters than those harboring SpWSD1, while recombinant yeast harboring ScWSD1-m with the mutant site T239S could synthesize more C34 esters than those harboring ScWSD1 (Fig. 6). These results indicate that the positive selection site can affect the wax ester profile.

## Discussion

Studying an alpine plant that survives under harsh environmental stresses, such as drought, is particularly useful because the plant has evolved complicated mechanisms against such environmental stresses (Byars *et al.*, 2007). There are differences in rainfall from east to west on the QTP owing to the westerly monsoon wind system, which causes average annual precipitation ranging from 20 mm to 600 mm (Liu and Chen, 2015). Along the moisture gradient, the distribution range of *S. capillacea* is from 300 mm to 600 mm in the northwestern QTP, while *S. purpurea*, a dominant grass in alpine arid and semi-arid meadows, is widely distributed, including in extremely arid areas (Fig. 1). Related species at different geographical locations may exhibit specific variations to adapt to the local environment. *Stipa capillacea* had shown lower resistance to extreme drought, and its distribution was mainly dependent on rainfall (Rychnovská, 1965). In this study, we examined the drought response between *S. capillacea* and *S. purpurea* by monitoring their physiological performances under drought conditions. Comparatively, *S. capillacea* showed lower water-holding capacities in leaves and photosynthesis capability during drought stress and re-watering (Fig. 2). Additionally, the higher survival rate of *S. purpurea* after re-watering was also investigated (Fig. 2c), and it was demonstrated to be a drought-tolerant plant (Yang *et al.*, 2015). *Stipa purpurea* grows in extremely dry biotopes, which indicates that its drought tolerance might result from long-term natural selection.

A fundamental goal of evolutionary biology is to identify and study the loci that contribute to key phenotypes involved in adaptation, reproduction, and survival. The analyses of full-length transcriptomes has assisted in revealing how variation in gene regulation is associated with phenotypic variation (Liu *et al.*, 2017; Xu *et al.*, 2017). Thus, we analyzed the changes in transcript expression in *S. capillacea* and *S. purpurea* during drought and re-watering using PacBio Iso-Seq and Illumina sequencing technologies (Fig. 3a). An enrichment analysis of differentially expressed genes predicted that cutin, suberin, and wax biosynthesis play critical roles in the drought response of the two *Stipa* species (Fig. 3b). Additionally, nine genes involved in wax synthesis had higher expression levels in both *Stipa* species after the drought treatment (Fig. 3c, d). These genes encoding wax biosynthetic enzymes, such as CER1, CER4, CER6, and CER10, were identified mainly through forward genetic approaches using a collection of wax-deficient Arabidopsis or maize (*Z. mays*) lines and their mutants (Rowland *et al.*, 2006; Samuels *et al.*, 2008; Islam *et al.*, 2009). Changes in the expression levels of wax biosynthetic genes may affect the biosynthesis of cuticular waxes during plant responses to drought (Zhou *et al.*, 2018; Patwari *et al.*, 2019).

Cuticular wax accumulation can reduce water loss in leaves by regulating non-stomatal transpiration (Riederer and Schreiber, 2001; Zhou *et al.*, 2018). In this study, the results of water loss and chlorophyll leaching off detached leaves in a dark environment also showed that cuticular wax played an important role in preventing water loss of *S. purpurea* leaves (Fig. 4). In addition, the main plant strategies to cope with drought stress under natural selection tend to involve avoiding dehydration rather than encouraging dehydration tolerance (Zhang *et al.*, 1997). However, it is difficult to explain why the leaf water content of *S. purpurea* is greater than that of *S. capillacea* after drought treatments, because the expression levels of the nine wax synthetic genes of *S. capillacea* were also up-regulated. Therefore, we performed a positive selection analysis of these nine genes and their orthologs from six closely related species. For these genes, we found strong evidence of positive selection at the T239 and A403 sites of WSD1 from subgroup 1 in a separate clade of the WSD family (Fig. 5). WSD1 is involved in wax ester synthesis, and the accumulation of wax esters increases in *A. thaliana* leaves and stems during drought (Li *et al.*, 2008; Patwari *et al.*, 2019). Here, we found that *S. purpurea* shows a greater accumulation of wax esters under normal water conditions, and an increase in wax esters was also observed during a water deficiency compared with wax ester levels in *S. capillacea* (Fig. 4). In addition, there were differences in the T239 site between SpWSD1 and ScWSD1 (Fig. 5c). We therefore focused on the effects of the SpWSD1 T239 site, with its positive selection signal, on wax synthesis.

Alpine plants have evolved various morphological and physiological characteristics to adapt to extreme environments on the QTP, and some genes and sites have been identified as being under positive selection pressure by genome/transcriptome sequencing (Qiao *et al.*, 2016; Zhang *et al.*, 2019). Most functional studies of positive selection sites focus on the influence of a predicted protein structure. In our study, the specific site mutants *SpWSD1-*m and *ScWSD1*-m were constructed. The activity levels of SpWSD1, ScWSD1, and the mutants were investigated by recombinant yeast H1246 expressing WSD1 with C16 and C18 as substrates. C34 could not be detected in yeast H1246 expressing the empty plasmid, which was used as a negative control. Four recombinant yeast H1246 lines independently heterologously expressed SpWSD1, SpWSD1-m, ScWSD1, and ScWSD1-m, and resulted in C34 wax ester formation, indicating that WSD1 from both *Stipa* species has the acyltransferase activity of WSD. Recently, the activity of AtWSD1, as a bifunctional WSD enzyme, when expressed in yeast was also reported (Li *et al.*, 2008).

In addition, mutational analyses of bacterial WSD (Ma1; WP_011783747) from *M. aquaeolei* VT8 also showed that mutations of 25G, 144 A, 356L, 360A, and 405M sites could alter substrate selectivity to synthesize wax esters of different lengths (Barney et al., 2013, 2015; Petronikolou *et al.*, 2018). However, 259T and 403A sites of SpWSD1 do not have corresponding positions in the Ma1 sequence due to absence of the corresponding structure Ma1 based on the pairwise alignment of the two gene sequences (Supplementary Fig. S6). Thus, differences in wax ester profiles based on positive selection at the T239 site of SpWSD1 may be beneficial to the response of *S. purpurea* to drought, indicating that *S. purpurea* has evolved specific strategies involving its wax biosynthetic pathway as part of its long-term adaptation to extreme drought conditions on the QTP.

## Supplementary data

Supplementary data are available at *JXB* online.

Fig. S1. The species distribution is shown as a percentage of the total homologous sequences with an E-value of at least 1.0E-5.

Fig. S2. Correlation analysis of the expression of two pairs from full-length transcripts.

Fig. S3. KEGG pathway analysis of differentially expressed genes of *S. capillacea* under drought conditions.

Fig. S4. Heat map of expression profiles (in log2-based RPKM) of wax synthesis pathway genes in *S. purpurea* and *S. capillacea* following exposure to drought stress.

Fig. S5. The stomatal morphology from detached leaves of SC and SP were detected for each sample after dark treatment.

Fig. S6. Sequence alignment of SpWSD1 (GenBank accession no. MK994184) and Ma1 from *M. aquaeolei* VT8 (GenBank accession no. WP_011783747) using the SWISS-MODEL server.

Table S1. Primers designed for qRT-PCR.

Table S2. Primers for site-directed mutagenesis of WSD1.

Table S3. Statistical summary of PacBio SMRT sequencing.

Table S4. Statistical summary of the *S. purpurea* and *S. capillacea* reads generated using the Illumina HiSeq 2000.

Table S5. Statistical summary of RNA-seq reads mapping to the full-length transcripts.

Table S6. WSD genes identified in two *Stipa* species and six relatives

Table S7. Parameters and log LRTs of site-specific models.

eraa194_suppl_Supplementary_Material

## Data Availability

The raw sequence data reported in this paper have been deposited in the Genome Sequence Archive in the BIG Data Center, Beijing Institute of Genomics (BIG), Chinese Academy of Sciences, under accession numbers CRA002020, CRA002019, CRA002018, and CRA002017 that are publicly accessible at http://bigd.big.ac.cn/gsa.
