# Supplementary material for: Uncovering the role of a positive selection site of wax ester synthase/diacylglycerol acyltransferase in two closely related Stipa species in wax ester synthesis under drought stress
Source: J Exp Bot. 2020 Apr 20;71(14):4159–70. doi: 10.1093/jxb/eraa194 (PMC7475244; doi:10.1093/jxb/eraa194)
Supplement: eraa194_suppl_Supplementary_Material [file eraa194_suppl_supplementary_material.pdf]

Uncovering the role of positive selection site of wax ester synthase/diacylglycerol acyltransferase in two closely related Stipa species (Stipa capillacea and Stipa purpurea) accessions in wax ester synthesis under drought stress

Running title: Effect of positive selection site of WSD1 gene on wax ester synthesis in Stipa species

Yunqiang Yang[1,2,3,a], Zhili Zhou[1,2,3,a], Yan Li[1,2,3,5], Yanqiu Lv[6], Danni Yang[1,2,3,5], Shihai Yang[1,2,3], Jianshuang Wu[4], Xiong Li[1,2,3], Zhijia Gu[1], Xudong Sun[1,2,3*], Yongping Yang[1,2,3*]

[1]Key Laboratory for Plant Diversity and Biogeography of East Asia, Kunming Institute of Botany, Chinese Academy of Science, Kunming, 650204, China

[2]Plant Germplasm and Genomics Center, Kunming Institute of Botany, Chinese Academy of Sciences, Kunming 650201, China

[3]Institute of Tibetan Plateau Research at Kunming, Kunming Institute of Botany, Chinese Academy of Sciences, Kunming, 650201, China

[4]Functional Biodiversity, Dahlem Center of Plant Sciences, Free University of Berlin, 14195 Berlin, Germany

[5]University of Chinese Academy of Sciences, Beijing, 100049, China

[6] College of Life Sciences, Changchun Normal University, Changchun, 130032, China

[a] These authors contributed equally to this work.

*Corresponding author:

Xudong Sun

Email: sunxundong@mail.kib.ac.cn

Phone: 86-871-65220873

Yongping Yang

Email: yangyp@mail.kib.ac.cn

Phone: 86-871-65223398

Supplemental information:

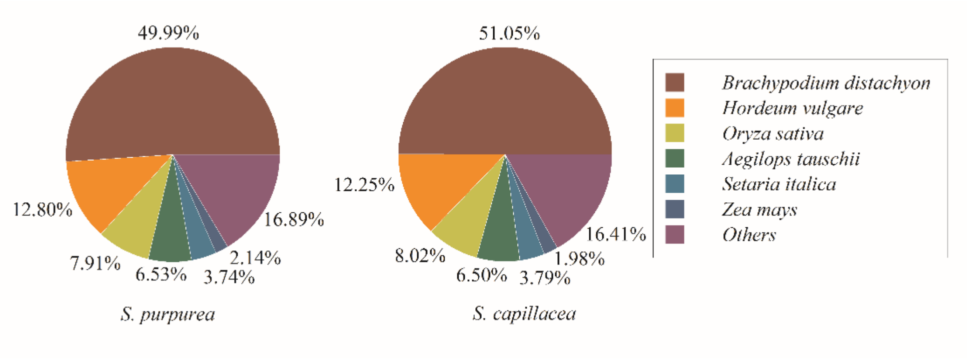

Supplemental Figure 1. The species distribution as a percentage of the total homologous sequences with E-values of at least 1.0E-5.

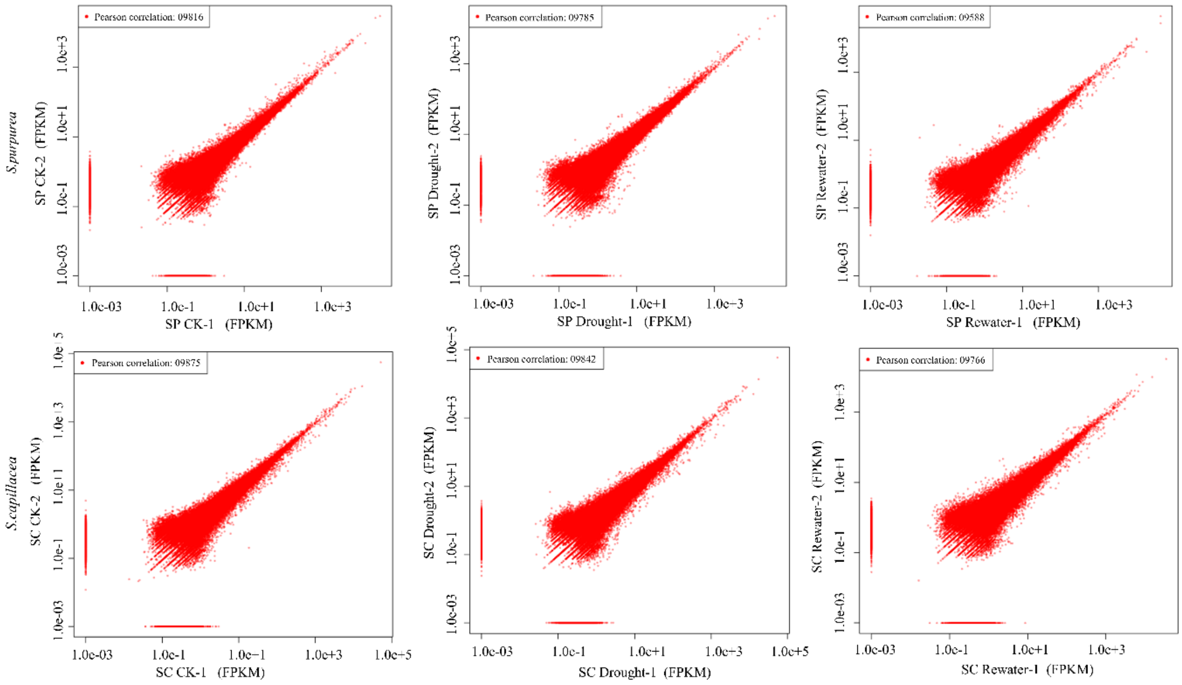

Supplemental Figure 2. Correlation analysis of genes differentially expressed between different treatments and their controls from full-length transcripts.

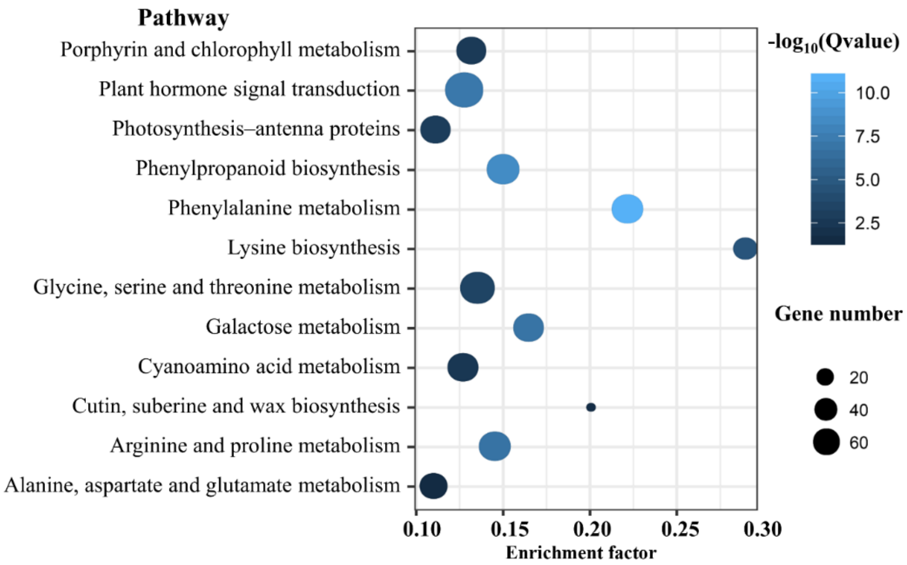

Supplemental Figure 3. KEGG pathway analysis of differentially expressed genes of *S. capillacea* under drought conditions compared with the control.

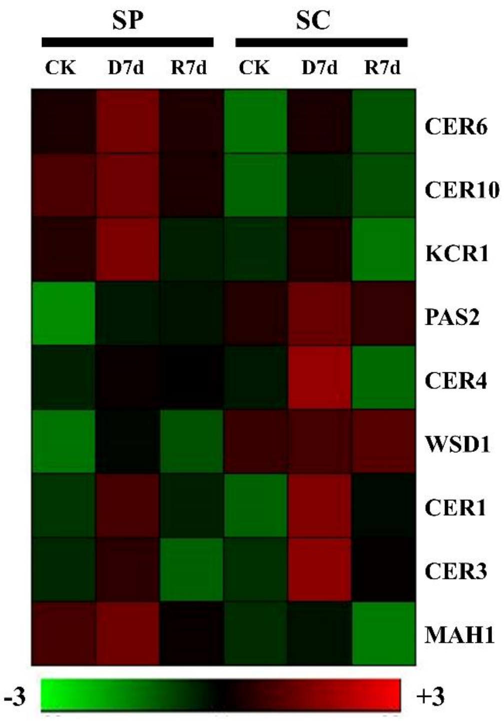

Supplemental Figure 4. Heat map of expression profiles (in log2-based FPKM) of wax synthetic pathway genes in *S. purpurea* and *S. capillacea* following exposure to drought stress.

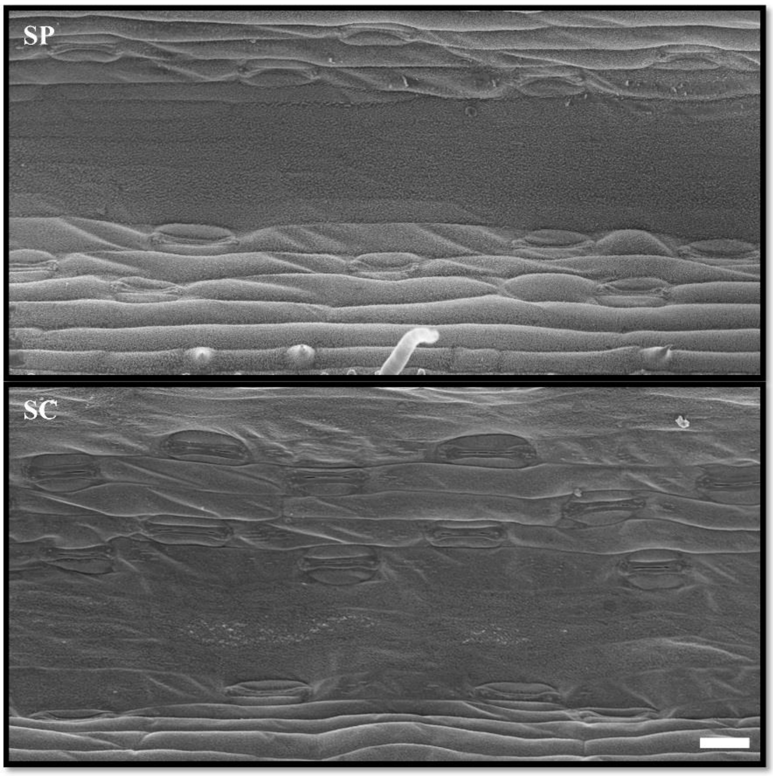

Supplemental Figure 5. The stomatal morphology from detached leaves of SC and SP were detected for each sample after dark treatment. Bar= 20 μm.

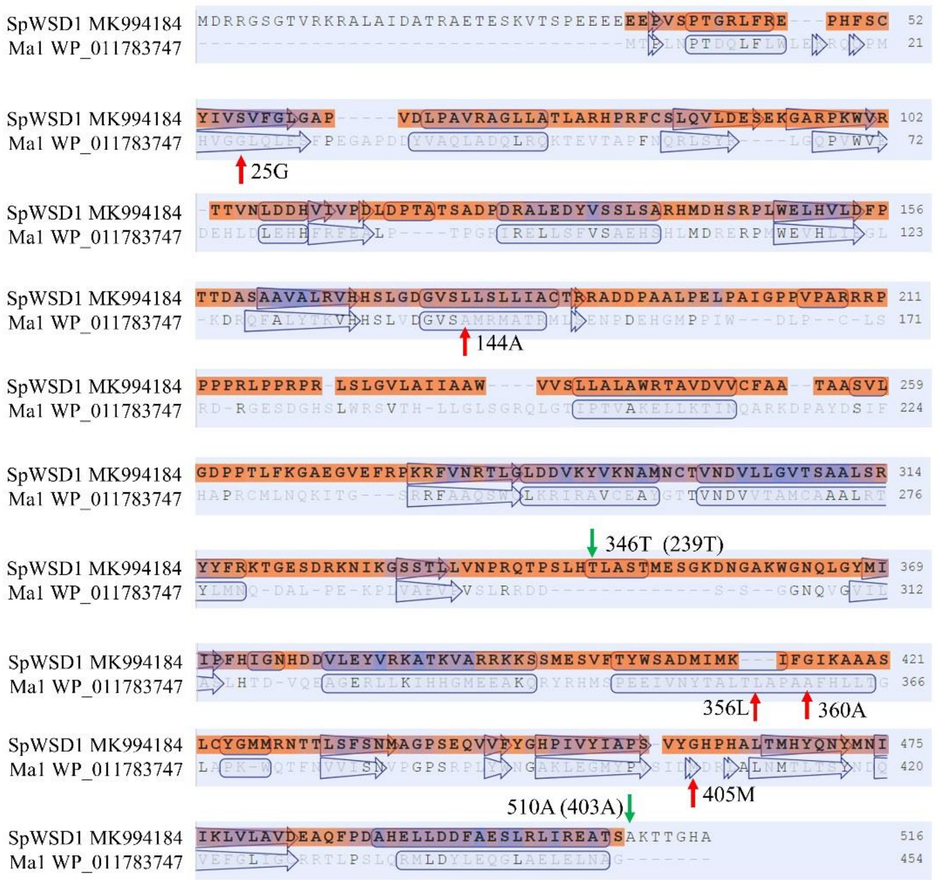

Supplemental Figure 6. Sequence alignment of SpWSD1 (GenBank accession number:MK994184) and Ma1 from *M. aquaeolei* VT8 (GenBank accession number: WP_011783747) using the SWISS-MODEL server. Some of the identified specific residues (Barney *et al*., 2013; Barney *et al*., 2015; Petronikolou *et al*., 2018) that can affect the substrate selection of the Ma1 were indicated by red arrows. 346T and 510A amino acid sites of SpWSD1, corresponding to positive selection sites 239T and 403A, respectively, were instructed to use green arrows.

Supplemental Table 1. Primers designed for qRT-PCR.

Gene name	*S.purpurea*		*S.capillacea*	
	Primer F (5’-3’)	Primer R (5’-3’)	Primer F (5’-3’)	Primer R (5’-3’)
CER6	CAACATCCGCAGCTTCAACC	AGTTGGGCGTGATGATCTCC	CTTCGGCTCCGGGTTTAAGT	TAGTGCTTGAGCACGTCGG
CER10	CTAAGGCGAGGTTCACCGTT	GTGGAGTAGCTCGATGGCTC	GAGCCATCGAGCTACTCCAC	ATGGCATAGCGAGAACGAGG
KCR1	TCCGATCCGCTCTACTCCAT	TTGCACATCAATGCCCTTGC	CACCTATGCTCGTGCTGCTA	CAGGCGCGTACTGTCTACAA
PAS2	TACTGCTCGTCGTTGTGCTT	GAGCTTGAGCCCTTGCAAAC	TGGGGAGATTCAATCGGTGC	CCTTGACCTGATTCGCTGGT
CER4	AAGGGTGGGCTAGGCAATTT	TGATGACAACCGGAAGGTCG	CATGCAACTCGTCGACCTCT	GGTCTTGGGGTCGAAGTTGA
WSD1	TGGTGTAACTTCTGCCGCAT	CGTCTGTCTTGGGTTGACGA	CTGCTACATCGTGTCCGTGT	GACTCGTCCAACACCTGGAG
CER1	CCCGAAGTTGGGGGTAAGAC	CACAAGCAATAGCACGAGCC	CCCTCCATTCTGTTGCGGAT	AGGATGATCTGGTCGTCCCA
CER3	TGAGCAAGCACCCTAACCTG	CGGTGAGGAACACCTCCTTC	CACGCATTTCCACCAGTTCG	ACACCCCTCTCCATCGTGTA
MAH1	GGCCGGCGACAAGATACTAA	TTCCTCGGTGATCCACCTCT	CCAACCTCCCTCACCTTCAC	ATGAAGACGTGGCGGACATT
Actin1	GCTGGATTCTGGAGATGGTGTC	TTACTCATTCACCACTACGGCTG	GATCCTGACTGAGCGTGGTT	GCTGGTCTTAGCAGTCTCCA

Supplemental Table 2. Primers for the site-directed mutagenesis of WSD1.

Gene name	Primer F (5'-3')	Primer R (5'-3')
SpWSD1	ATGGATCGCCGCGGCAGCGGCAC	TGCGTGTCCTGTGGTTTTCGCTG
ScWSD1	ATGGATCACCGCGGCAGCGGCA	TGCGTGTCCTGTGGTTTTCGCT
SpWSD1-m	AACCCAAGACAGACGCCTAGCCTGCATTCATTGGCTA	CCTTGCCGGATTCCATCGTACTAGCCAATGAATGCAG
ScWSD1-m	AACCTAAGACAGACGCCTGGCCTGCATACATTGGCTA	CCTTGCCGGATTCCATCATACTAGCCAATGTATGCAG

Supplemental Table 3. Statistical summary of PacBio SMRT sequencing.

Samples	SP			All	SC			All
cDNA Size	1-2K	2-3K	3-6K		1-2K	2-3K	3-6K	
SMRT Cells	2	2	1		2	2	1	
Reads of Insert	143,242	137,067	57,445	337,754	129,059	146,200	59,931	335,190
Number of five prime reads	85,174	74,576	32,393	192,143	57,864	74,486	35,180	167,530
Number of three prime reads	94,635	82,418	33,857	210,910	68,715	82,087	36,758	187,560
Number of poly-A reads	91,937	80,867	33,553	206,357	64,724	79,840	36,237	180,801
Number of filtered short reads	17,568	13,237	1,346	32,151	23,607	15,776	1,956	41,339
Number of non-full-length reads	52,694	62,988	29,073	144,755	59,248	71,390	28,930	159,568
Number of full-length reads	72,980	60,842	27,026	160,848	46,204	59,034	29,045	134,283
Number of full-length non-chimeric reads	72,726	60,728	26,999	160,453	45,781	58,874	29,013	133,668
Average full-length non-chimeric read length	1,350	2,217	3,503	7,070	1,152	2,268	3,369	6,789
Full-Length Percentage (FL%)	50.95%	44.39%	47.05%	100.00%	35.80%	40.38%	48.46%	100.00%
Artificial Concatemers（%）	0.35%	0.19%	0.10%	0.64%	0.92%	0.27%	0.11%	1.30%

Supplemental Table 4. Statistical summary of the *S. purpurea* and *S. capillacea* reads generated using Illumina HiSeq 2000.

Samples ID	Read Sum	Base Sum	GC(%)	Q30(%)
SP CK -1	20597917	4119583400	54.3	93.03
SP CK -2	27056702	5411340400	53.54	91.17
SP Drought -1	31269863	6253972600	55.22	90.36
SP Drought -2	26803921	5360784200	53.58	90.87
SP Rewater -1	30400831	6080166200	54.57	90.85
SP Rewater -2	28961564	5792312800	53.54	91.5
SC CK -1	27950361	5590072200	54.86	90.81
SC CK -2	30489118	6097823600	53.69	91.41
SC Drought -1	23415769	4683153800	54.7	91.43
SC Drought -2	23369795	4673959000	53.16	91.2
SC Rewater -1	29482702	5896540400	53.34	90.95
SC Rewater -2	27932291	5586458200	52.81	91.56

Supplemental Table 5. Statistical summary of RNA-Seq reads mapped to the full-length transcripts.

	S. purpurea		*S.capillacea*	
CK-1	Reads number	Percentage	Reads number	Percentage
Total Reads	20597917	100%	27950361	100%
Mapped Reads	17122548	83.13%	23309312	83.40%
Uniq mapped Reads	2423556	14.15%	3020261	12.96%
Multi mapped Reads	14698992	85.85%	20289051	87.04%
CK-2				
Total Reads	27056702	100%	30489118	100%
Mapped Reads	22391201	82.76%	24891416	81.64%
Uniq mapped Reads	3246098	14.50%	3279030	13.17%
Multi mapped Reads	19145103	85.50%	21612386	86.83%
D7d-1				
Total Reads	31269863	100%	23415769	100%
Mapped Reads	25428644	81.32%	19044438	81.33%
Uniq mapped Reads	3855911	15.16%	2910319	15.28%
Multi mapped Reads	21572733	84.84%	16134119	84.72%
D7d-2				
Total Reads	26803921	100%	23369795	100%
Mapped Reads	21600359	80.59%	18488449	79.11%
Uniq mapped Reads	3413952	15.81%	2834429	15.33%
Multi mapped Reads	18186407	84.19%	15654020	84.67%
R7d-1				
Total Reads	30400831	100%	29482702	100%
Mapped Reads	25440854	83.68%	24954581	84.64%
Uniq mapped Reads	3333330	13.10%	2871222	11.51%
Multi mapped Reads	22107524	86.90%	22083359	88.49%
R7d-2				
Total Reads	28961564	100%	27932291	100%
Mapped Reads	23726951	81.93%	22840503	81.77%
Uniq mapped Reads	3392697	14.30%	3012354	13.19%
Multi mapped Reads	20334254	85.70%	19828149	86.81%

Supplemental Table 6. WSD genes identified in two Stipa species and six relatives

**Name**	**GeneBank Protein ID**
SpWSD1	MK994184
ScWSD1_	MK994185
Bradi2g46640.2	XP_014753790
HORVU3Hr1G062900.2	BAJ93204
AET3Gv20642100.1	XP_020162382
LOC_Os01g48874.1	XP_01562148
Seita.5G271700.1	XP_004969548
GRMZM2G077375_P01	XP_008675193
Bradi2g17077.1	XP_010231047
AET1Gv20924400.2	No Found
Seita.3G152100.1	XP_004961273
LOC_Os05g48260.1	XP_015637860
SP.PB15825	MN952987
AET1Gv20059400.1	XP_020162351
Bradi3g18630.1	XP_014756310
Bradi3g18636.3	PNT66934
Seita.2G020900.1	XP_004955390
HORVU3Hr1G002050.2	BAJ93623
HORVU7Hr1G024590.1	BAK05274
SC.PB10917	MN952986
SP.PB17174	MN952988
HORVU5Hr1G082160.1	XP_020163719
HORVU5Hr1G082210.6	KAE8804413
AET5Gv20742100.24	XP_020163713
Seita.5G332500.1	XP_004970103
LOC_Os01g56370.1	BAF06289
LOC_Os01g56360.1	XP_015621333
AET6Gv20026000.2	XP_020163013
HORVU6Hr1G013350.2	KAE8769450
SP.PB21665	MN952989
AET3Gv20715700.6	XP_020175550
Bradi2g51150.3	PNT72937
Seita.9G398600.1	XP_004984321
Seita.8G209100.1	RCV39261
Seita.8G209200.1	XP_004979823

Supplemental Table 7. Parameters and log likelihood ratio tests of site-specific models.

Sub group	2△/M2avsM1a	2△/M7vsM8[a]	M8estimates[b]	Positive selected site(posterior>90)[c]
1	3.1268	11.6948**	p0= 0.99372 p= 0.62131 q= 2.30598 p1=0.00628 ω=3.66627	239T 403A
2	0	10.1497**	p0= 0.90654 p= 0.77513 q= 1.50409 p1=0.09346 ω=1.38390	427H
3	0	16.3556**	p0= 0.88483 p= 0.99657 q= 4.66605 p1=0.11517 ω=1.18402	None
4	0	15.0835**	p0= 0.86893 p= 1.05245 q= 3.02611 p1=0.13107 ω=1.00000	373 S
All	0	11.7531**	p0= 0.92064 p= 0.97655 q= 2.73674 p1=0.07936 ω=1.00000	384A 385 K

a. *: significant at 5% level, **: significant at 1% level.

b. ω is dN:dS estimated under M8; p1 is the inferred proportion of positively selected sites.

c. Sites potentially under positive selection identified under model M8 are listed according to conserved sequence numbering. Positively selected sites with posterior probability >0.95 are bolded.
